# eHealth versus face-to-face support for remission of type 2 diabetes by calorie restriction (eHealth DIabetes remission Trial): study protocol for a non-inferiority parallel group randomised controlled trial

**DOI:** 10.1136/bmjopen-2024-095100

**Published:** 2025-07-22

**Authors:** Julia Otten, Anna Tellström, Claudia Schien, Youssef Chninou, Lars Lindholm, Anna Winkvist, Per Liv, Andreas Stomby

**Affiliations:** 1 Department of Public Health and Clinical Medicine, Umeå University, Umea, Sweden; 2 Clinical Research Centre, Umeå University Hospital, Umeå, Sweden; 3 Department of Epidemiology and Global Health, Umeå University, Umeå, Sweden; 4 Department of Internal Medicine and Clinical Nutrition, Goteborgs Universitet, Goteborg, Sweden; 5 Department of Health, Medicine and Caring Sciences, Linköping University, Linköping, Sweden

**Keywords:** eHealth, Diabetes Mellitus, Type 2, Obesity

## Abstract

**Introduction:**

If a person is in diabetes remission, even if only for a short time, this reduces the risk of later diabetes complications and lowers healthcare costs. A recent study shows that long-term remission of type 2 diabetes can be achieved through calorie restriction using total diet replacement. However, this intervention involves support through face-to-face meetings every 2 to 4 weeks over a 2-year period, which is not feasible in routine care with limited resources. Therefore, we have developed an eHealth programme to help patients achieve diabetes remission through calorie restriction in a cost-effective manner. Our primary hypothesis is that an eHealth programme will be non-inferior to face-to-face meetings in helping patients with type 2 diabetes achieve remission through caloric restriction. Our second hypothesis is that eHealth support will be more cost-effective than face-to-face support.

**Methods and analysis:**

The eHealth DIabetes remission Trial is a multicentre, two-arm, non-inferiority, open-label, randomised controlled parallel group trial with blinded endpoint assessment conducted at two centres in Sweden. The study duration is 2 years. People with type 2 diabetes (≤6 years duration) use total diet replacement (approximately 900 kcal/day) with the aim of losing 15 kg and achieving diabetes remission. Participants are randomly assigned to either the eHealth support group or the face-to-face support group. The treatment programme to achieve and maintain weight loss is the same in both groups, but the method of support differs between the groups. The primary outcome is haemoglobin A1c (HbA1c) after 1 year. The secondary outcome is HbA1c at 6 months and 2 years. Other important secondary outcomes are diabetes remission rate, body weight and cost-effectiveness. The latter is assessed using the incremental cost per quality-adjusted life-years gained.

**Ethics and dissemination:**

The study was approved by the Swedish Ethical Review Authority (Dnr 2022-02242-01, 2023-03707-02). The results will be published in peer-reviewed scientific journals and discussed at national and international conferences and with patient organisations.

**Trial registration number:**

ClinicalTrials.gov (NCT05491005).

**STRENGTHS AND LIMITATIONS OF THIS STUDY**

- A strength of the study is its comparison of eHealth-based calorie restriction for diabetes remission with an active comparator (face-to-face support), rather than with standard care alone.
- Another strength is that the eHealth programme, specifically developed for this trial, is integrated into the national medical platform ‘Stöd och behandling’ on 1177.se, making it accessible to healthcare providers across Sweden from study start.
- A limitation of the study is the use of a wider non-inferiority margin for HbA1c of 8 mmol/mol (0.8%), compared with the narrower margin of 4 mmol/mol (0.4%) commonly employed in pharmaceutical trials.

## Introduction

 A breakthrough in the treatment of type 2 diabetes is the recent Diabetes REmission Clinical Trial (DiRECT), which shows that long-term remission of type 2 diabetes can be achieved through calorie restriction.^1 2^ The study found that of the people with type 2 diabetes who received total diet replacement with 850 kcal/day over a period of 3 to 5 months, 46% were in diabetes remission after 1 year, and 36% were still in remission after 2 years.^2^ Diabetes remission is defined as haemoglobin A1c (HbA1c) below 48 mmol/mol (6.5%) without diabetes medication.^3^ The HbA1c value reflects the average plasma glucose level of the last 2 months. In the DiRECT study, the diabetes remission rate was up to 70% in those who had maintained a weight loss of at least 15 kg.^2^ Remarkably, the main motivator for study participants to lose weight was to achieve diabetes remission and thus avoid diabetes medication,^4^ that is, remission was a much greater source of motivation than the promise that weight loss would reduce the risk of future complications and increase life expectancy.

According to a health economic evaluation of the DiRECT study, diabetes remission with total diet replacement is cost-saving after 5–6 years despite face-to-face meetings between patient and healthcare provider every 2 to 4 weeks.^5^ Based on the results of the DiRECT study, calorie restriction with the goal of 15 kg weight loss and diabetes remission is now strongly recommended in the treatment guidelines of the European and American Diabetes Associations.^6^

As far as we know, no dietary intervention with the overarching aim of remission of type 2 diabetes has yet been conducted comparing eHealth with face-to-face care. eHealth can have many benefits: It may be more effective than face-to-face care because it eliminates travel time for the patient, and it allows for a more flexible schedule for both the patient and the healthcare provider. In studies where eHealth was used to lower blood glucose levels in people with diabetes, eHealth was superior to conventional care.^7^ In addition, a meta-analysis found that prevention of type 2 diabetes through eHealth via weight loss was successful in 63% of interventions in the short term and in 21% in the long term, that is, 12 months or longer.^8^ However, only half of the studies were randomised controlled trials, and the control group usually received no intervention. The question of whether an intervention with eHealth support is more effective than face-to-face support is therefore still open.

### Unmet need

Although recommended in the guidelines, significant weight loss and remission of type 2 diabetes through calorie restriction is not commonly used in routine care.^6^ A major reason for this is the challenge of achieving and maintaining weight loss over time without intensive and long-term support from care providers, who have limited resources. eHealth can offer an effective and potentially more patient-friendly approach than face-to-face meetings, as it is less time-consuming than standard care for both the patient and the healthcare provider. In a randomised trial, we aim to investigate the clinical effectiveness and cost-effectiveness of achieving diabetes remission through calorie restriction with a specially developed eHealth programme compared with face-to-face meetings. The comparison group in this study receives an intervention identical to the intervention in the DiRECT study, as this intervention, which is based on face-to-face counselling, has been shown to be effective. The intervention group in this study participates in a similar programme, but the support is provided via an eHealth platform.

### Objective

Our primary hypothesis is that eHealth support is non-inferior to face-to-face support in helping patients with type 2 diabetes achieve remission through calorie restriction. Our secondary hypothesis is that eHealth support will be more cost-effective than face-to-face support.

## Methods and analysis

### Trial design and setting

The eHealth DIabetes remission Trial (eDIT) is a multicentre, two-arm, non-inferiority, open-label, randomised controlled parallel-group trial with blinded endpoint assessment. The study is being conducted at two centres in Sweden: Umeå and Jönköping. In Umeå, both the intervention sessions and the outcome measurement visits will take place at the Clinical Research Centre at Umeå University Hospital. In Jönköping, the intervention visits will take place at Ryhov County Hospital’s Rehabilitation Centre and the outcome measurement visits will take place at Bra Liv Råslätt Primary Healthcare Centre.

This paper adheres to the 2013 SPIRIT guidelines for reporting a clinical trial protocol. A SPIRIT checklist can be found in online supplemental material 1.^9 10^

### Recruitment and eligibility

Participants are recruited through advertisement in local newspapers and social media as well as personal invitations from diabetes nurses. Recruitment started in Umeå in August 2022 and in Jönköping in November 2023.

### Inclusion criteria

- Type 2 diabetes with a duration of 6 years or less (diagnosis based on two documented diagnostic tests of either HbA1c, fasting blood glucose or 2-hour blood glucose after an oral glucose tolerance test (OGTT) using WHO diagnostic criteria).^11^
- Age 20–70 years.
- Body mass index (BMI)≥27 kg/m^2^.
- HbA1c≥43 mmol/mol (6.1%) at baseline if taking glucose lowering medication.
- HbA1c≥48 mmol/mol (6.5%) at baseline if not on glucose lowering medication.

### Exclusion criteria

- Insulin treatment.
- Weight loss of more than 5 kg in the last 6 months.
- Diagnosed eating disorder.
- Chronic kidney disease stage 4 or 5 (estimated glomerular filtration rate (eGFR) <30 mL/min/m^2^).
- Myocardial infarction in the last 6 months.
- Severe heart failure defined as equivalent to New York Heart Association (NYHA) grade III or IV.
- Ongoing cancer.
- Pregnancy.
- Treatment with antipsychotic medication.

### Randomisation

After the initial examination, participants are randomised in a 1:1 ratio to either the eHealth support group or the face-to-face support group (table 1). Randomisation is performed using permuted blocks with random sizes of 2, 4 or 6 and is stratified by study centre. The allocation sequence is generated by the statistician PL and uploaded to the *Research Electronic Data Capture (REDCap*). On completion of all baseline assessments, a nurse retrieves the participant’s group assignment from *REDCap* and notifies the participant.

**Table 1:** Timetable of enrolment, interventions and assessments for the eHealth DIabetes remission Trial (eDIT)

Timepoint	Study period					
	Enrolment	Allocation	Post-allocation			
	-t_1_	0	Diet start	6 months	12 months	24 months
Enrolment						
Eligibility screen	X					
Informed consent	X					
Allocation		X				
Interventions						
eHealth support group			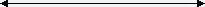			
Face-to-face support group			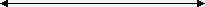			
All participants: Weight loss intervention			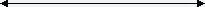			
Assessments						
HbA1c	X			X	X	X
Anthropometry*	X			X	X	X
Blood pressure	X			X	X	X
Plasma lipid profile and liver enzymes	X			X	X	X
Fasting glucose and OGTT	X			X	X	X
Questionnaires†	X			X	X	X
Health economic evaluation						X

This table is adapted from the SPIRIT 2013 statement.^9^

* Anthropometry = body weight, waist circumference, hip circumference, abdominal height.

† Questionnaires: EuroQol Quality - 5 Dimensions - 5 Levels (EQ-5D-5L), Brunnsviken Brief Quality of Life Scale, 36-Item Short Form Survey (SF-36), three factor eating questionnaire, Karolinska Exhaustion Disorder Scale and food frequency questionnaire 2020.

HbA1c, haemoglobin A1c; OGTT, oral glucose tolerance test.

### Intervention

The main goal is a permanent remission of diabetes (HbA1c <48 mmol/mol (6.5%) without diabetes medication). Participants are randomly assigned to either the eHealth support group or the face-to-face support group. The structured weight management programme, which includes a phase of total diet replacement followed by a gradual reintroduction of food, dietary counselling and behaviour change strategies to achieve and maintain weight loss, is the same in both groups, but the method of support differs between the groups (eHealth vs face-to-face). The dietary counselling is based on the European recommendations for the dietary management of diabetes, the Nordic Nutrition Recommendations 2012 and the Swedish Food Agency’s dietary guidelines from 2015.^12^,^14^

#### Total diet replacement phase (weeks 0–12)

The study participants in both the eHealth support group and the face-to-face support group replace all food with the total diet replacement formula Modifast (Navamedic AB, Sävedalen, Sweden) for 3 months (table 1). The total diet replacement contains about 900 kcal/day. The aim is to lose 15 kg of weight in these 12 weeks. If this goal is not achieved after 3 months, the study participants are recommended to extend the total diet replacement for additional weeks. The total diet replacement formula is commercially available and is paid for by the study participants themselves at a 20% reduced price.

#### Food reintroduction phase (weeks 13–18)

After 3 months of total diet replacement and reaching the weight goal, food is gradually reintroduced for 6 weeks to reduce the risk of regaining weight. One meal at a time is reintroduced over about 2 weeks. During this time, participants receive guidance on how to prepare nutritious, low-calorie meals and snacks that meet the dietary recommendations. To allow flexibility for participants, the food reintroduction phase can be longer or shorter than 6 weeks.

#### Weight maintenance phase (weeks 19–104)

In this phase, the focus is on maintaining the lower body weight by working on regular meal patterns and goal setting, planning healthy meals, limiting high calorie foods, increasing physical activity, self-monitoring behaviours and dealing with challenges and setbacks. If weight regain occurs during the weight maintenance phase, a rescue plan with diet replacement is recommended, that is, either a period of total diet replacement or replacement of one or two meals a day. Treatment with Orlistat is offered to all participants experiencing difficulty in maintaining weight loss. Despite their potent weight-reducing effects, GLP1 receptor agonists and tirzepatide are not used for weight loss purposes, as they would compromise the ability to determine diabetes remission. However, these medications may be initiated if blood glucose levels rise, as described in the following paragraph.

#### Medication discontinuation

Glucose-lowering medication is discontinued under the supervision of the study physician when total diet replacement is started. In most patients with short-term diabetes, blood glucose levels improve within a week if they adhere to the total diet replacement.^1^ In addition, diabetes remission can only be diagnosed if the patient is without such medication.^3^ However, if blood glucose levels rise, glucose-lowering medication is reintroduced (online supplemental material 2). All anti-hypertensive medication and diuretics are discontinued when starting the total diet replacement, as many people suffer from hypotension and orthostasis after starting a very low-calorie diet. However, participants do not discontinue blood pressure medications taken for reasons other than hypertension (eg, beta-blockers used to treat heart disease are not discontinued). Based on blood pressure measurements during the study, medication will be restarted if blood pressure rises (online supplemental material 3), following criteria adapted from the DiRECT study.^15^

#### Treatment programme

The intervention in this study is delivered by a registered dietitian, a nurse and a physician using a detailed intervention protocol that ensures a comparable intervention regardless of staff category (online supplemental materials 4 and 5). At both study sites, most video and physical meetings are conducted by the dietitian. Participants meet the study staff individually and no group sessions are offered. To maintain a lower body weight, participants use a support programme based on cognitive behavioural therapy and other behaviour change strategies. These include goal setting, action planning, problem solving, eating behaviour change techniques (eg, a regular eating pattern), stimulus control, self-efficacy, self-monitoring of behaviour, strategies for dealing with setbacks and regaining weight, and education about healthy eating habits. This is an evidence-based strategy for weight maintenance after weight loss.^16 17^

The therapy programme is identical for the eHealth support group and the face-to-face support group and contains the following main sections: (1) introduction and goals; (2) find your inner motivation; (3) treatment with the diet replacement formula; (4) food reintroduction; (5) physical activity; (6) goal setting; (7) maintaining a lower body weight; (8) eating behaviours; (9) dealing with setbacks; and (10) carbohydrates, fats and special treats. Each main section of the programme has between four and 16 subsections. In addition, the programme contains 14 homework assignments, for example, ‘my goals’, ‘eating habits I want to change’, ‘switch to more healthy fats’ and ‘plan your special treats’. All participants receive feedback on their homework from the study dietitian, nurse or doctor. Each study participant is offered the *GRV Fitness Tracker S1* pedometer (GRV media, Widnes, England, UK) at the start of the food reintroduction phase.

#### eHealth support group

All study information and the treatment programme are provided via a digital application and video meetings (online supplemental material 6). The digital programme is available on the Swedish national healthcare platform *1177.se* and contains the information and homework of the therapy programme. The participant has access to the digital application via a mobile phone or PC. New chapters of the therapy programme are made available to the participant during the scheduled video sessions (online supplemental material 4). Participants are equipped with the Omron HN289 scale (Omron Healthcare Co Ltd, Kyoto, Japan), the Accu-Check Instant blood glucose metre (Roche Diabetes Care GmbH, Mannheim, Germany) and the Omron M3 Comfort blood pressure monitor (Omron Healthcare Co Ltd, Kyoto, Japan). Participants measure their body weight, fasting blood glucose and blood pressure every morning and record this on the digital platform. A member of the study team evaluates all daily measurements once a week and gives each participant individual feedback via a message in the digital programme. The study participants can send messages to the study team via this digital platform and receive a response once a week. Participants in the eHealth group have eleven scheduled video support meetings with the study dietitian, study nurse or study doctor (online supplemental material 4). If the daily home measurements show that a participant is not achieving the intended weight loss or maintenance, written feedback is provided by study personnel via the programme’s messaging function. This feedback includes personalised information, such as dietary guidance or a contingency plan involving total diet replacement. Participants are encouraged to respond in order to facilitate the development of a mutually agreed-upon plan. If resolution cannot be achieved through messaging alone, additional contact may be initiated through supplementary video sessions between study staff and the participant. Every second month, study participants measure the HbA1c values at home and send them by post to the laboratory at Umeå University Hospital. The HbA1c result is reported back to the participant via a message in the digital application.

#### Face-to-face support group

The support for the face-to-face group follows the protocol of the DiRECT study (online supplemental material 6): Participants have an appointment at the office with the study doctor/nurse/dietician every 2 weeks for the first 5 months and thereafter every 4 weeks until the end of the study.^18^ This means 33 scheduled face-to-face meetings and additional appointments are possible. At each visit, participants measure their body weight with the Omron HN289 scale (Omron Healthcare Co Ltd, Kyoto, Japan), non-fasting blood glucose with the Accu-Chek Instant blood glucose metre (Roche Diabetes Care GmbH, Mannheim, Germany) and blood pressure with the Omron M3 Comfort blood pressure monitor (Omron Healthcare Co Ltd, Kyoto, Japan) under the supervision of the study staff. The therapy programme for the face-to-face support group is identical to that of the eHealth support group, but the programme is carried out during the face-to-face appointments (online supplemental material 5). Participants receive the chapters of the therapy programme and the homework in paper form at the face-to-face meetings. The HbA1c level is measured at the study visits after 6, 12 and 24 months and communicated to the participant at the following face-to-face appointment (table 1).

### Primary outcome

The primary outcome is HbA1c as a continuous outcome at 12 months.

### Secondary outcomes

1. The primary outcome HbA1c as a continuous outcome at 6 and 24 months.
2. Incremental costs per quality-adjusted life-years (QALY) gained (at 24 months only).
3. Incremental costs per diabetes remission (at 24 months only).
4. Estimated lifetime costs (at 24 months only).
5. Diabetes remission rate (proportion of participants with HbA1c<48 mmol/mol (<6.5%) without diabetes medication).
6. Body weight and BMI.
7. Reduction in body weight by at least 15 kg (proportion of participants).
8. Waist circumference.
9. Abdominal height.
10. Blood pressure measured at the research facilities.
11. 24 hours blood pressure home monitoring.
12. Plasma lipid profile.
13. Plasma liver enzymes.
14. Fasting blood glucose.
15. Plasma glucose 120 min after the OGTT.
16. Daily steps (measured with activity tracker).
17. Quality of life (EuroQol Quality - 5 Dimensions - 5 Levels (EQ-5D-5L)).
18. Quality of life (Brunnsviken Brief Quality of Life Scale).
19. Estimation of own health (36-Item Short Form Survey (SF-36)).
20. Relation to food (three-factor eating questionnaire).
21. Estimation of exhaustion (Karolinska Exhaustion Disorder Scale).
22. Eating habits (food frequency questionnaire 2020).
23. Study experience (written answers to open questions, at 24 months only).
24. Number of prescribed antidiabetic medications.
25. Number of prescribed antihypertensive medication.
26. Number of participants that discontinue the intervention.
27. Long-term follow-up after study completion of HbA1c, body weight, blood pressure, diabetes complications, and usage of antidiabetic and blood pressure medication.

All secondary outcomes are assessed at 6, 12 and 24 months if not otherwise stated above. A description of other pre-specified outcomes can be found on *ClinicalTrials.gov* (NCT05491005).

### Publication plan

We plan to publish the primary outcome once all participants have completed their 12-month follow-up. The article will include the secondary outcomes described above at 6 and 12 months, with the exception of the health economic analysis (secondary outcomes 2, 3 and 4), which will be published as a separate article after the 24-month follow-up.

### Study procedure and data collection

The same study protocol is used in both study centres. At each study centre, a team consisting of a dietician, nurse and doctor carries out the intervention. The doctors involved in the study are a resident physician in endocrinology, a specialist in general medicine, an endocrinologist and a house officer. The same study staff carries out the intervention in both the eHealth support group and the face-to-face support group. Due to the nature of the study, neither the participants nor the study team are blinded to the intervention. However, the analyses and interpretation of the results are carried out blinded to group allocation.

Adherence to the assigned intervention is monitored by recording all contacts between participants and study personnel (eg, face-to-face meetings, video meetings, messages, digitally reported weight measurements). If participants do not adhere to the study schedule, they are asked to continue with the intervention (by phone for the face-to-face support group and by digital message for the eHealth support group). It is not possible to switch from the eHealth support group to the face-to-face support group or vice versa. However, if a participant is unable to fulfil the study schedule, the frequency of support meetings or measurements can be adjusted. The study team makes every effort to ensure that participants complete the study or at least the outcome measures. If a study participant drops out of the study, he/she is asked for a final measurement of weight and HbA1c.

The study personnel assume responsibility for the participants’ diabetes and blood pressure medication for the duration of the study. However, it is not discouraged that participants contact their usual healthcare providers regarding their diabetes or other health problems. Any damages resulting directly from participation in the study are covered by the regional healthcare provider’s patient insurance. Due to the open study design and the relatively small size of the study, there is no data monitoring committee for this study. No interim analysis is planned before analysing the primary outcome after 12 months. Should the study protocol be modified, the principal investigators are responsible for communicating any significant changes to study participants, study personnel and the trial registry.

Participants’ data during the intervention are collected and managed using *REDCap*, which is hosted at Umeå University.^19 20^ *REDCap* is a secure, web-based software platform developed to support data collection for research studies. All participant data in *REDCap* are coded, and the code key is kept in a secure location by the responsible researchers. The data management plan for this study is available online on a platform provided by the Umeå University Library.^21^

Anyone interested in participating in the study has an appointment for a telephone interview with a member of the study staff. If the person fulfils the inclusion criteria and is willing to take part in the study, an appointment is made with one of the study physicians, during which the consent form is signed. A sample of the consent form is provided in online supplemental material 7.

At the beginning and after 6, 12 and 24 months, the study participants undergo the following examinations (table 1): Body weight is measured to the nearest 100 g using a Tanita WB-100 MA scale (Tanita Cooperation, Tokyo, Japan) at the Umeå study site and a SECA 704 scale (seca, Hamburg, Germany) at the Jönköping study site while fasting, wearing light clothing and no shoes. One kilogramme is deducted for clothing. Height is measured to the nearest mm with a Seca 264 at the Umeå study site and with a Seca 216 (both seca, Hamburg, Germany) at the Jönköping study site. Waist circumference is measured to the nearest mm at the end of a normal exhalation with a tape measure in the horizontal plane midway between the iliac crest and the lowest rib. The abdominal height is measured in the supine position as the distance between the highest point of the abdomen and the hard surface below it after a normal exhalation using a sagittal abdominal measuring device (AJ Medical, Lidingö, Sweden). Blood pressure at the research facilities is measured on the right upper arm after 5 min of rest in a sitting position using a Welch Allyn 3400 blood pressure monitor (Welch Allyn, Skaneateles Falls, USA). Blood pressure is measured three times, and the average of the last two measurements is reported. Home blood pressure monitoring is performed over a 24-hour period using the OnTrak device (Spacelabs Healthcare, Snoqualmia, WA, USA). Venous blood samples for HbA1c, fasting glucose, plasma lipid profile and plasma liver enzymes are analysed at the hospital laboratory closest to the study site. Additionally, venous blood samples are collected in the fasting state and at 30, 60, 90 and 120 min following the OGTT. These samples are stored at −80°C for future, as yet unspecified, analyses. Data regarding daily steps will be collected using the GRV fitness tracker non-Bluetooth fitness watch (GRV, USA). Participants answer the EQ-5D-5L and the Brunnsviken Brief Quality of Life Scale to assess their quality of life,^22 23^ the SF-36 questionnaire to assess their health,^24^ the 21-item three-factor eating questionnaire to investigate their relation to food,^25^ the Karolinska Exhaustion Disorder Scale to estimate exhaustion^26^ and the validated food frequency questionnaire 2020 to assess their eating habits.^27^ At the end of the study, participants answer 15 open questions about their study experience. All questionnaires and questions are answered directly in *REDCap* using a tablet, smartphone or computer.

An OGTT is performed, for which the participants take 75 g of glucose dissolved in water (Top Star 75, Esteriplas, Feira, Portugal, total volume 200 mL). Before taking the glucose and 120 min afterwards, blood glucose is measured from venous blood and immediately analysed with Hemocue 201 RT (HemoCue AB, Ängelholm, Sweden). Participants are advised to refrain from strenuous physical activity the day before the OGTT. They are advised to eat a standardised meal at 7 pm the day before the test. For this meal, participants are offered two different simple recipes containing 660 kcal with 90 g of carbohydrates, 23–28 g of protein and 18–20 g of fat. The OGTT is performed at 8 am after a 12-hour overnight fast (only tap water is allowed; no caffeine or smoking). Participants are advised to skip taking medication in the morning and to travel to the clinic by car or bus to minimise physical activity.

All diseases and abnormalities in the test results are enquired about at each intervention visit (both during video sessions and face-to-face meetings) and recorded in *REDCap*. In addition, possible side effects to total diet replacement are actively enquired about and also recorded in *REDCap*.

Because long-term data on diabetes remission by total diet replacement are lacking, study participants will be followed after they have finished the study. Data on HbA1c, body weight, blood pressure, diabetes complications, and usage of anti-diabetic and blood pressure medication will be obtained from the national diabetes registry and the healthcare systems computer system once per year. Data from the national diabetes registry from participants of this study will be compared with other patients in the national diabetes registry not participating in this study but matched for age, sex and diabetes duration to the study participants.

### Sample size and justification

The primary outcome HbA1c after 1 year is used to test whether eHealth support is non-inferior to face-to-face support. The non-inferiority margin (delta) for HbA1c is set at 8 mmol/mol (0.8%). This margin is based on the clinically relevant HbA1c reduction leading to a lower risk of microvascular complications in patients with newly diagnosed type 2 diabetes.^28^ This margin is further justified by the fact that commonly used glucose-lowering medications, such as SGLT2 inhibitors and metformin, typically achieve a mean HbA1c reduction of at least 8 mmol/mol compared with placebo,^29^ which means that a declaration of non-inferiority implies that eHealth support would have a non-zero effect. In the DiRECT study, the mean HbA1c level after 1 year was 50.6 mmol/mol (6.8%) with a SD of 13.3 mmol/mol (1.2%).^18^ If we assume the same SD in the present study, we need to randomise 96 study participants to achieve sufficient power in a test procedure based on the estimation of a CI based on the t-distribution as calculated with the *Sealed Envelope* online calculator.^30^ In the DiRECT study, 22% of participants dropped out of the intervention, but only 8% had HbA1c levels unavailable after 1 year. We therefore expect a drop-out rate of 10% and plan to include 106 participants in our study. For the calculation, we used a significance level (alpha) of 0.05 and a target power (1-beta) of 90%.

The main objective of this randomised trial is to demonstrate the non-inferiority of eHealth support compared with face-to-face support. HbA1c is a relevant surrogate measure for diabetes remission, and we therefore believe that HbA1c as a continuous variable rather than diabetes remission as a dichotomous outcome represents an appropriate trade-off between the project requirements and the costs and benefits for participants to investigate the non-inferiority of eHealth support compared with face-to-face support.

### Statistical analyses

For the primary outcome, the continuous variable HbA1c is analysed for non-inferiority using ANCOVA, with HbA1c at 12 months as the dependent variable and the group as the independent variable. The analysis will be adjusted for baseline HbA1c and study centre to account for stratified randomisation. The non-inferiority margin is 8 mmol/mol (0.8%). If a participant is taking medication to lower blood glucose, that participant’s HbA1c will be adjusted for the average blood glucose-lowering effect of the medication used, according to the meta-analysis by Tsapas and colleagues.^29^ The analysis is performed according to the intention-to-treat principle with imputation of missing values using multivariate imputation by chained equations.^31^ The same analysis will be performed on the full analysis set, that is, all randomised participants with HbA1c measurements at baseline and 12 months, as a sensitivity analysis. The intention-to-treat population is defined as all study participants that were randomised.

The secondary outcome diabetes remission in per cent will be tested for non-inferiority with a non-inferiority margin of 10 percentage points, followed by a test for superiority. The other secondary outcomes will be tested for superiority only.

For the cost-effectiveness analysis, the incremental costs per QALYs gained is the main outcome. Costs include the total diet replacement portions used, meetings with the study team including travel time, time taken by the study team to assess and provide feedback on participants’ measurements each week, other healthcare visits and hospital days, blood glucose-lowering and blood pressure-lowering medications, costs of HbA1c analyses and scales, blood pressure monitors and blood glucose metres. Participants will answer the SF-36 and EQ-5D-5L questionnaires to analyse how they experience their health and quality of life. Costs are analysed based on the intention-to-treat principle. Mean costs are calculated for each group, with cluster-adjusted standard errors for each cost item. Incremental costs per QALY after 2 years are given as the difference in total costs of the groups after 2 years divided by the difference in QALYs. The cost per QALY gained is compared with the Swedish threshold of SEK 500 000. If the ratio is below the threshold, the intervention is categorised as cost-effective. If both interventions are cost-effective, the most effective one is recommended for introduction into normal healthcare.

## Ethics and dissemination

The eDIT was approved by the Swedish Ethical Review Authority (Dnr 2022-02242-01) on 10 May 2022. All participants give written informed consent before participating in the study. This study is conducted on the basis of the Declaration of Helsinki and in accordance with Good Clinical Practise. The sponsor of the study is Umeå University. The two principal investigators, JO and AS, have access to the final data set and will conduct the analyses together with other researchers and research students. The results will be published in peer-reviewed journals by the principal investigators together with other researchers who fulfil the four author criteria of the International Committee of Medical Journal Editors.^32^ In addition, the results are presented at national and international scientific conferences and discussed at joint meetings with patient organisations. Participant-level data cannot be made publicly available due to legal regulations but may be obtained from the principal investigator on reasonable request.

## Supplementary material

10.1136/bmjopen-2024-095100 online supplemental file 1
