# Supplementary material for: eHealth versus face-to-face support for remission of type 2 diabetes by calorie restriction (eHealth DIabetes remission Trial): study protocol for a non-inferiority parallel group randomised controlled trial
Source: BMJ Open. 2025 Jul 22;15(7):e095100. doi: 10.1136/bmjopen-2024-095100 (PMC12306309; doi:10.1136/bmjopen-2024-095100)
Supplement: online supplemental file 1 [file bmjopen-15-7-s001.pdf]

Supplemental material 1 SPIRIT 2013 checklist

SPIRIT

STANDARD PROTOCOL ITEMS: RECOMMENDATIONS FOR INTERVENTIONAL TRIALS

Section/item	Item No	Description	Addressed on page number
**Administrative information**			
Title	1	Descriptive title identifying the study design, population, interventions, and, if applicable, trial acronym	_______1______
Trial registration	2a	Trial identifier and registry name. If not yet registered, name of intended registry	_______1______
	2b	All items from the World Health Organization Trial Registration Data Set	_______1-10____
Protocol version	3	Date and version identifier	_______1______
Funding	4	Sources and types of financial, material, and other support	_______10_____
Roles and responsibilities	5a	Names, affiliations, and roles of protocol contributors	_______1,10____
	5b	Name and contact information for the trial sponsor	_______9______
	5c	Role of study sponsor and funders, if any, in study design; collection, management, analysis, and interpretation of data; writing of the report; and the decision to submit the report for publication, including whether they will have ultimate authority over any of these activities	_______10_____
	5d	Composition, roles, and responsibilities of the coordinating centre, steering committee, endpoint adjudication committee, data management team, and other individuals or groups overseeing the trial, if applicable (see Item 21a for data monitoring committee)	_______8______

**Introduction**			
Background and rationale	6a	Description of research question and justification for undertaking the trial, including summary of relevant studies (published and unpublished) examining benefits and harms for each intervention	______3______
	6b	Explanation for choice of comparators	______3,4_____
Objectives	7	Specific objectives or hypotheses	______3______
Trial design	8	Description of trial design including type of trial (eg, parallel group, crossover, factorial, single group), allocation ratio, and framework (eg, superiority, equivalence, noninferiority, exploratory)	______4______
**Methods: Participants, interventions, and outcomes**			
Study setting	9	Description of study settings (eg, community clinic, academic hospital) and list of countries where data will be collected. Reference to where list of study sites can be obtained	______4______
Eligibility criteria	10	Inclusion and exclusion criteria for participants. If applicable, eligibility criteria for study centres and individuals who will perform the interventions (eg, surgeons, psychotherapists)	______4______
Interventions	11a	Interventions for each group with sufficient detail to allow replication, including how and when they will be administered	______4-7_____
	11b	Criteria for discontinuing or modifying allocated interventions for a given trial participant (eg, drug dose change in response to harms, participant request, or improving/worsening disease)	______5______
	11c	Strategies to improve adherence to intervention protocols, and any procedures for monitoring adherence (eg, drug tablet return, laboratory tests)	______7______
	11d	Relevant concomitant care and interventions that are permitted or prohibited during the trial	______8______
Outcomes	12	Primary, secondary, and other outcomes, including the specific measurement variable (eg, systolic blood pressure), analysis metric (eg, change from baseline, final value, time to event), method of aggregation (eg, median, proportion), and time point for each outcome. Explanation of the clinical relevance of chosen efficacy and harm outcomes is strongly recommended	______7,8_____
Participant timeline	13	Time schedule of enrolment, interventions (including any run-ins and washouts), assessments, and visits for participants. A schematic diagram is highly recommended (see Figure)	___Table 1,2___

Sample size	14	Estimated number of participants needed to achieve study objectives and how it was determined, including clinical and statistical assumptions supporting any sample size calculations	______8,9_____
Recruitment	15	Strategies for achieving adequate participant enrolment to reach target sample size	______4______
**Methods: Assignment of interventions (for controlled trials)**			
Allocation:			
Sequence generation	16a	Method of generating the allocation sequence (eg, computer-generated random numbers), and list of any factors for stratification. To reduce predictability of a random sequence, details of any planned restriction (eg, blocking) should be provided in a separate document that is unavailable to those who enrol participants or assign interventions	______4______
Allocation concealment mechanism	16b	Mechanism of implementing the allocation sequence (eg, central telephone; sequentially numbered, opaque, sealed envelopes), describing any steps to conceal the sequence until interventions are assigned	______4______
Implementation	16c	Who will generate the allocation sequence, who will enrol participants, and who will assign participants to interventions	______4______
Blinding (masking)	17a	Who will be blinded after assignment to interventions (eg, trial participants, care providers, outcome assessors, data analysts), and how	______7______
	17b	If blinded, circumstances under which unblinding is permissible, and procedure for revealing a participant's allocated intervention during the trial	______N/A____
**Methods: Data collection, management, and analysis**			
Data collection methods	18a	Plans for assessment and collection of outcome, baseline, and other trial data, including any related processes to promote data quality (eg, duplicate measurements, training of assessors) and a description of study instruments (eg, questionnaires, laboratory tests) along with their reliability and validity, if known. Reference to where data collection forms can be found, if not in the protocol	______8______
	18b	Plans to promote participant retention and complete follow-up, including list of any outcome data to be collected for participants who discontinue or deviate from intervention protocols	______7______

Data management	19	Plans for data entry, coding, security, and storage, including any related processes to promote data quality (eg, double data entry; range checks for data values). Reference to where details of data management procedures can be found, if not in the protocol	______8______
Statistical methods	20a	Statistical methods for analysing primary and secondary outcomes. Reference to where other details of the statistical analysis plan can be found, if not in the protocol	______9______
	20b	Methods for any additional analyses (eg, subgroup and adjusted analyses)	______9______
	20c	Definition of analysis population relating to protocol non-adherence (eg, as randomised analysis), and any statistical methods to handle missing data (eg, multiple imputation)	______9______
**Methods: Monitoring**			
Data monitoring	21a	Composition of data monitoring committee (DMC); summary of its role and reporting structure; statement of whether it is independent from the sponsor and competing interests; and reference to where further details about its charter can be found, if not in the protocol. Alternatively, an explanation of why a DMC is not needed	______8______
	21b	Description of any interim analyses and stopping guidelines, including who will have access to these interim results and make the final decision to terminate the trial	______7______
Harms	22	Plans for collecting, assessing, reporting, and managing solicited and spontaneously reported adverse events and other unintended effects of trial interventions or trial conduct	______8______
Auditing	23	Frequency and procedures for auditing trial conduct, if any, and whether the process will be independent from investigators and the sponsor	______8______
**Ethics and dissemination**			
Research ethics approval	24	Plans for seeking research ethics committee/institutional review board (REC/IRB) approval	______9______
Protocol amendments	25	Plans for communicating important protocol modifications (eg, changes to eligibility criteria, outcomes, analyses) to relevant parties (eg, investigators, REC/IRBs, trial participants, trial registries, journals, regulators)	______8______

Consent or assent	26a	Who will obtain informed consent or assent from potential trial participants or authorised surrogates, and how (see Item 32)	______8______
	26b	Additional consent provisions for collection and use of participant data and biological specimens in ancillary studies, if applicable	______8______
Confidentiality	27	How personal information about potential and enrolled participants will be collected, shared, and maintained in order to protect confidentiality before, during, and after the trial	______8______
Declaration of interests	28	Financial and other competing interests for principal investigators for the overall trial and each study site	______10_____
Access to data	29	Statement of who will have access to the final trial dataset, and disclosure of contractual agreements that limit such access for investigators	______9______
Ancillary and post-trial care	30	Provisions, if any, for ancillary and post-trial care, and for compensation to those who suffer harm from trial participation	______8______
Dissemination policy	31a	Plans for investigators and sponsor to communicate trial results to participants, healthcare professionals, the public, and other relevant groups (eg, via publication, reporting in results databases, or other data sharing arrangements), including any publication restrictions	______9,10____
	31b	Authorship eligibility guidelines and any intended use of professional writers	______9,10____
	31c	Plans, if any, for granting public access to the full protocol, participant-level dataset, and statistical code	______10_____
**Appendices**			
Informed consent materials	32	Model consent form and other related documentation given to participants and authorised surrogates	______8______
Biological specimens	33	Plans for collection, laboratory evaluation, and storage of biological specimens for genetic or molecular analysis in the current trial and for future use in ancillary studies, if applicable	______9______

Supplemental material 2 Reintroduction of glucose-lowering medication

A. Based on symptoms and/or capillary blood sugar
   - Not earlier than two weeks after the participant has started with total diet replacement.
   - If random blood sugar is above 20 mmol/L or if the participant experiences polyuria/thirst.
   - Discussion with the participant whether there is any other help that can be offered to successfully follow the diet plan with total diet replacement.
   - If weight reduction is as planned but blood glucose is still high: consider reintroduction of blood glucose-lowering medication. Start at the lowest dose and increase gradually.
   - Previously used glucose lowering medication is started according to the following ranking order:
     1. Metformin
     2. SGLT2 inhibitors
     3. GLP1 receptor agonists (including tirzepatide)
     4. DDP4 inhibitors
     5. Sulfonylurea/glinides
     6. Pioglitazone

B. Based on HbA1c
   - eHealth support group: in that group, HbA1c is checked with home measurements every two months. If HbA1c ≥ 53 mmol/mol (7.0%) on two repeated HbA1c measurements, blood sugar-lowering medication is reintroduced according to the ranking order above. After HbA1c ≥ 53 mmol/mol (7.0%) has been measured once, the participant is encouraged to lose weight.
   - Face-to-face support group: this groups checks HbA1c only at baseline, 6 months, 12 months and 24 months. If HbA1c ≥ 53 mmol/mol (7.0%) has been measured at the 6-months or the 12-months visit, an extra HbA1c is checked two months later. If HbA1c ≥ 53 mmol/mol (7.0%) the second time, blood sugar-lowering medication is reintroduced according to the ranking order above. After HbA1c ≥ 53 mmol/mol (7.0%) has been measured once, the participant is encouraged to lose weight.

Supplemental material 3 Reintroduction of blood pressure medication

- Inform the study participant to decrease salt intake.
- If systolic blood pressure is above 140 mmHg on repeated measurements or if diastolic blood pressure is above 90 mmHg on repeated measurements, previously used blood pressure medication is started according to the following ranking order:
  1. Angiotensin receptor blockers
  2. Angiotensin-converting enzyme (ACE) inhibitors
  3. Calcium channel blockers
  4. Spironolactone
  5. Beta blockers

6. Thiazides
7. Alpha blockers
8. All other blood pressure medications

- eHealth support group: if systolic blood pressure is above 140 mmHg or diastolic above 90 mmHg at most of the reported measurements during a week, blood pressure-lowering medication is started or increased.
- Face-to-face support group: if systolic blood pressure is above 140 or diastolic above 90 mmHg at two consecutive face-to-face appointments at the clinic, blood pressure lowering medication is started or increased.
- During the first two study weeks systolic blood pressure needs to be above 165 mmHg or diastolic above 105 mmHg to start blood pressure medication.
- The systolic and diastolic values are assessed separately: if one day only systolic and the next day only diastolic blood pressure is elevated, blood pressure medication is not increase.
- If necessary, every other week blood pressure medication is increased, or an additional medication is added.

Supplemental material 4 Therapy program for the eHealth support group delivered at the 11 video meetings

- Video meeting week 0
  - General information about the therapy program
  - Information on daily measurement of body weight, fasting blood sugar and blood pressure that is reported daily on the digital platform
  - Decision on the starting date for the total diet replacement
  - In the digital program the following modules are made available for the study participant (and discussed at the video meeting):
    - Goals of the therapy program
    - Introduction to the therapy program
    - My motivation
    - Eight steps to changing lifestyle habits
    - Daily measurements of body weight, fasting glucose and blood pressure
    - Introduction to diet replacement
    - What happens in the body when you use total diet replacement
    - Possible side effects of total diet replacement
    - How to deal with feelings of hunger
    - Diet replacement with diet replacement formula Modifast®
  - Presenting homework 1 “My inner motivation”
- Video meeting week 2
  - Follow up of weight loss, fasting blood sugar, blood pressure and the intervention in general
  - Follow up homework 1 “My inner motivation”

- <u>Video meeting week 6</u>
  - Follow up of weight loss, fasting blood sugar, blood pressure and the intervention in general
- <u>Video meeting week 12</u>
  - Follow up of weight loss, fasting blood sugar, blood pressure and the intervention in general
  - Diet and weight history (causes of weight problems, daily routines, eating habits, obstacles to change, social support)
  - In the digital program the following modules are made available to the study participant (and discussed at the video meeting):
    - Stepwise reintroduction of food
    - Breakfast
    - About maintaining weight
    - Keyhole labeling and food labeling
    - Meal ideas: breakfast menu
  - Presenting homework 2a "Planning my breakfast for a week"
  - Presenting homework 2b "Evaluation of my breakfast planning"
- <u>Video meeting week 14</u>
  - Follow up of weight loss and weight maintenance, fasting blood sugar, blood pressure and the intervention in general
  - Follow up homework 2a "Planning my breakfast for a week"
  - Follow up homework 2b "Evaluation of my breakfast planning"
  - The step counter *GRV Fitness Tracker S1* (GRV) is sent to the participant.
  - In the digital program the following modules are made available to the study participant (and discussed at the video meeting)
    - Regular physical activity
    - All steps count
    - Healthy lunch and dinner including lunch and dinner menus
    - Vegetables
    - Protein
    - Carbohydrates
    - Fats
  - Presenting homework 3a "Estimating my physical activity"
  - Presenting homework 3b "My step count"
- <u>Video meeting week 16</u>
  - Follow up of weight loss and weight maintenance, fasting blood sugar, blood pressure and the intervention in general
  - Follow up homework 3a "Estimating my physical activity"
  - Follow up homework 3b "My step count"
  - In the digital program the following modules are made available to the study participant (and discussed at the video meeting)
    - Healthy snacks
    - How can you deal with sauces?

        - Calorie reduced diet plan with recommended kcal/day
        - Goal setting
    - Presenting homework 4 “My goals”
    - Presenting homework 5 “Plan number of steps per day”
- Video meeting week 18
    - Follow up of weight loss and weight maintenance, fasting blood sugar, blood pressure and the intervention in general
    - Follow up homework 4 “My goals”
    - Follow up homework 5 “Plan number of steps per day”
    - The participant is offered orlistat as treatment if he/she is interested.
    - In the digital program the following modules are made available to the study participant (and discussed at the video meeting):
        - Why is it difficult to maintain weight after weight loss?
        - Factors that facilitate long-term weight maintenance
        - Healthy eating habits (regular meals, healthy eating pattern, finding your way)
    - Presenting homework 6 “My regular meals”
    - Presenting homework 7 “Eating habits I want to change”
- Video meeting week 20
    - Follow up of weight loss and weight maintenance, fasting blood sugar, blood pressure and the intervention in general
    - Follow up homework 6 “My regular meals”
    - Follow up homework 7 “Eating habits I want to change”
    - In the digital program the following modules are made available to the study participant (and discussed at the video meeting):
        - Hunger and cravings
        - Why is it easy to overeat?
        - It’s not your fault
        - Awareness and planning are required to change habits
        - This may increase your food cravings
        - How to handle food triggers in your environment
        - Coping with emotions using food
        - Sleeping difficulties and stress
        - Keeping a food diary
    - Presenting homework 8 “My food diary”
    - Presenting homework 9 “My challenges when changing eating habits”
- Video meeting week 26
    - Follow up of weight loss and weight maintenance, fasting blood sugar, blood pressure and the intervention in general
    - Follow up homework 8 “My food diary”
    - Follow up homework 9 “My challenges when changing eating habits”

    - In the digital program the following modules are made available to the study participant (and discussed at the video meeting)
        - Setback – an opportunity to learn something new
        - Automatic and conscious thoughts
        - Thinking traps (replacing dysfunctional thoughts)
    - Presenting homework 10a “Evaluate a setback”
    - Presenting homework 10b “How to handle a setback”
    - Presenting homework 11 “My thinking traps”
- Video meeting week 38
    - Follow up of weight loss and weight maintenance, fasting blood sugar, blood pressure and the intervention in general
    - Follow up homework 10a “Evaluate a setback”
    - Follow up homework 10b “How to handle a setback”
    - Follow up homework 11 “My thinking traps”
    - In the digital program the following modules are made available to the study participant (and discussed at the video meeting):
        - What is the function of carbohydrates in the body?
        - Foods rich in carbohydrates
        - “Slow” and “fast” carbohydrates
        - Dietary fibers
        - Fruit, vegetables and legumes
        - Whole grains
        - Fats
        - Calorie-rich and nutrient-low foods
        - Plan your special treats
        - Beverages containing sugar
        - Alcohol
    - Presenting homework 12a “Increasing dietary fibers in my food”
    - Presenting homework 12b “Evaluating the assignment of increasing dietary fiber”
    - Presenting homework 13a “Switch to more healthy dietary fats”
    - Presenting homework 13b “Evaluating the assignment of switching to more healthy dietary fats”
    - Presenting homework 14 “Planning your special treats”
- Video meeting week 54
    - Follow up of weight loss and weight maintenance, fasting blood sugar, blood pressure and the intervention in general
    - Follow up homework 12a “Increasing dietary fibers in my food”
    - Follow up homework 12b “Evaluate the assignment of increasing dietary fiber”
    - Follow up homework 13a “Switch to more healthy dietary fats”
    - Follow up homework 13b “Evaluate the assignment of switching to more healthy dietary fats”

    - Follow up homework 14 “Planning your special treats”

Supplemental material 5 Therapy program for the face-to-face support group delivered at 33 appointments

- Face-to-face appointment week 0
    - General information about the therapy program
    - Nurse or physician teaches the participant to measure body weight, non-fasting blood glucose and blood pressure himself/herself in the consultation room
    - Decision on the starting date for the total diet replacement
    - Written and oral information on the following topics of the therapy program:
        - Goals of the therapy program
        - Introduction to the therapy program
        - My motivation
        - Eight steps to changing lifestyle habits
        - Introduction to diet replacement
        - What happens in the body when you use total diet replacement?
        - Possible side effects of total diet replacement
        - How to deal with feelings of hunger
        - Diet replacement with diet replacement formula Modifast®
    - Presenting homework 1 “My inner motivation”
- Face-to-face appointment week 1
    - Participant measures body weight, non-fasting blood glucose and blood pressure himself/herself
    - Follow up of weight loss, blood sugar, blood pressure and the intervention in general
    - Follow up homework 1 “My inner motivation”
- Face-to-face appointment week 2
    - Participant measures body weight, non-fasting blood glucose and blood pressure himself/herself
    - Follow up of weight loss, blood sugar, blood pressure and the intervention in general
- Face-to-face appointment week 4
    - Participant measures body weight, non-fasting blood glucose and blood pressure himself/herself
    - Follow up of weight loss, blood sugar, blood pressure and the intervention in general
- Face-to-face appointment week 8
    - Participant measures body weight, non-fasting blood glucose and blood pressure himself/herself
    - Follow up of weight loss, blood sugar, blood pressure and the intervention in general
- Face-to-face appointment week 10
    - Participant measures body weight, non-fasting blood glucose and blood pressure himself/herself

    - Follow up of weight loss, blood sugar, blood pressure and the intervention in general
- Face-to-face appointment week 12
    - Participant measures body weight, non-fasting blood glucose and blood pressure himself/herself
    - Follow up of weight loss and weight maintenance, blood sugar, blood pressure and the intervention in general
    - Diet and weight history (causes of weight problems, daily routines, eating habits, obstacles to change, social support)
    - Written and oral information on the following topics of the therapy program:
        - Stepwise reintroduction of food
        - Breakfast
        - About maintaining weight
        - Keyhole labeling and food labeling
        - Meal ideas: breakfast menu
    - Presenting homework 2a "Planning my breakfast for a week"
    - Presenting homework 2b "Evaluation of my breakfast planning"
- Face-to-face appointment week 14
    - Participant measures body weight, non-fasting blood glucose and blood pressure himself/herself
    - Follow up of weight loss and weight maintenance, blood sugar, blood pressure and the intervention in general
    - Follow up homework 2a "Planning my breakfast for a week"
    - Follow up homework 2b "Evaluation of my breakfast planning"
    - The participant receives the step counter *GRV Fitness Tracker S1* (GRV).
    - Written and oral information on the following topics of the therapy program:
        - Regular physical activity
        - All steps count
        - Healthy lunch and dinner including lunch and dinner menus
        - Vegetables
        - Protein
        - Carbohydrates
        - Fats
    - Presenting homework 3a "Estimating my physical activity"
    - Presenting homework 3b "My step count"
- Face-to-face appointment week 16
    - Participant measures body weight, non-fasting blood glucose and blood pressure himself/herself
    - Follow up of weight loss and weight maintenance, blood sugar, blood pressure and the intervention in general
    - Follow up homework 3a "Estimating my physical activity"
    - Follow up homework 3b "My step count"
    - Written and oral information on the following topics of the therapy program:

        - Healthy snacks
        - How can you deal with sauces?
        - Calorie reduced diet plan with recommended kcal/day
        - Goal setting
    - Presenting homework 4 “My goals”
    - Presenting homework 5 “Plan number of steps per day”
- Face-to-face appointment week 18
    - Participant measures body weight, non-fasting blood glucose and blood pressure himself/herself
    - Follow up of weight loss and weight maintenance, blood sugar, blood pressure and the intervention in general
    - The participant is offered orlistat as treatment if he/she is interested.
    - Follow up homework 4 “My goals”
    - Follow up homework 5 “Plan number of steps per day”
    - Written and oral information on the following topics of the therapy program:
        - Why is it difficult to maintain weight after weight loss?
        - Factors that facilitate long-term weight maintenance
        - Healthy eating habits (regular meals, healthy eating pattern, finding your way)
    - Presenting homework 6 “My regular meals”
    - Presenting homework 7 “Eating habits I want to change”
- Face-to-face appointment week 20
    - Participant measures body weight, non-fasting blood glucose and blood pressure himself/herself
    - Follow up of weight loss and weight maintenance, blood sugar, blood pressure and the intervention in general
    - Follow up homework 6 “My regular meals”
    - Follow up homework 7 “Eating habits I want to change”
    - Written and oral information on the following topics of the therapy program
        - Hunger and cravings
        - Why is it easy to overeat?
        - It’s not your fault
        - Awareness and planning are required to change habits
        - This may increase your food cravings
        - How to handle food triggers in your environment
        - Coping with emotions using food
        - Sleeping difficulties and stress
        - Keeping a food diary
    - Presenting homework 8 “My food diary”
- Face-to-face appointment week 22

    - Participant measures body weight, non-fasting blood glucose and blood pressure himself/herself
    - Follow up of weight loss and weight maintenance, blood sugar, blood pressure and the intervention in general
    - Follow up homework 8 “My food diary”
    - Presenting homework 9 “My challenges when changing eating habits”
- <u>Face-to-face appointment week 26</u>
    - Participant measures body weight, non-fasting blood glucose and blood pressure himself/herself
    - Follow up of weight loss and weight maintenance, blood sugar, blood pressure and the intervention in general
    - Follow up homework 9 “My challenges when changing eating habits”
    - Written and oral information on the following topics of the therapy program:
        - Setback – an opportunity to learn something new
    - Presenting homework 10a “Evaluate a setback”
    - Presenting homework 10b “How to handle a setback”
- <u>Face-to-face appointment week 30</u>
    - Participant measures body weight, non-fasting blood glucose and blood pressure himself/herself
    - Follow up of weight loss and weight maintenance, blood sugar, blood pressure and the intervention in general
    - Follow up homework 10a “Evaluate a setback”
    - Follow up homework 10b “How to handle a setback”
    - Written and oral information on the following topics of the therapy program:
        - Automatic and conscious thoughts
        - Thinking traps (replacing dysfunctional thoughts)
    - Presenting homework 11 “My thinking traps”
- <u>Face-to-face appointment week 34</u>
    - Participant measures body weight, non-fasting blood glucose and blood pressure himself/herself
    - Follow up of weight loss and weight maintenance, blood sugar, blood pressure and the intervention in general
    - Follow up homework 11 “My thinking traps”
    - Written and oral information on the following topics of the therapy program:
        - What is the function of carbohydrates in the body?
        - Foods rich in carbohydrates
        - “Slow” and “fast” carbohydrates
        - Dietary fibers
        - Fruit, vegetables and legumes
        - Whole grains
    - Presenting homework 12a “Increasing dietary fibers in my food”

    Presenting homework 12b “Evaluating the assignment of increasing dietary fiber”
- <u>Face-to-face appointment week 38</u>

    - Participant measures body weight, non-fasting blood glucose and blood pressure himself/herself
    - Follow up of weight loss and weight maintenance, blood sugar, blood pressure and the intervention in general
    - Follow up homework 12a “Increasing dietary fiber in my food”
    - Follow up homework 12b “Evaluate the assignment of increasing dietary fiber”
    - Written and oral information on the following topics of the therapy program:
        - Fats
    - Presenting homework 13a “Switch to more healthy dietary fats”
    - Presenting homework 13b “Evaluate the assignment of switching to more healthy dietary fats”
- <u>Face-to-face appointment week 42</u>
    - Participant measures body weight, non-fasting blood glucose and blood pressure himself/herself
    - Follow up of weight loss and weight maintenance, blood sugar, blood pressure and the intervention in general
    - Follow up homework 13a “Switch to more healthy dietary fats”
    - Follow up homework 13b “Evaluate the assignment of switching to more healthy dietary fats”
    - Written and oral information on the following topics of the therapy program
        - Calorie-rich and nutrient-low food
        - Plan your special treats
        - Beverages containing sugar
        - Alcohol
    - Presenting homework 14 “Planning your special treats”
- <u>Face-to-face appointment week 46</u>
    - Participant measures body weight, non-fasting blood glucose and blood pressure himself/herself
    - Follow up of weight loss and weight maintenance, blood sugar, blood pressure and the intervention in general
    - Follow up homework 14 “Planning your special treats”
- <u>Face-to-face appointment week 50</u>
    - Participant measures body weight, non-fasting blood glucose and blood pressure himself/herself
    - Follow up of weight loss and weight maintenance, blood sugar, blood pressure and the intervention in general
- <u>Face-to-face appointment week 54</u>
    - Participant measures body weight, non-fasting blood glucose and blood pressure himself/herself
    - Follow up of weight loss and weight maintenance, blood sugar, blood pressure and the intervention in general
- <u>Face-to-face appointment week 58</u>
    - Participant measures body weight, non-fasting blood glucose and blood pressure himself/herself
    - Follow up of weight loss and weight maintenance, blood sugar, blood pressure and the intervention in general
- <u>Face-to-face appointment week 62</u>
    - Participant measures body weight, non-fasting blood glucose and blood pressure himself/herself
    - Follow up of weight loss and weight maintenance, blood sugar, blood pressure and the intervention in general

- Face-to-face appointment week 66
  - Participant measures body weight, non-fasting blood glucose and blood pressure himself/herself
  - Follow up of weight loss and weight maintenance, blood sugar, blood pressure and the intervention in general
- Face-to-face appointment week 70
  - Participant measures body weight, non-fasting blood glucose and blood pressure himself/herself
  - Follow up of weight loss and weight maintenance, blood sugar, blood pressure and the intervention in general
- Face-to-face appointment week 74
  - Participant measures body weight, non-fasting blood glucose and blood pressure himself/herself
  - Follow up of weight loss and weight maintenance, blood sugar, blood pressure and the intervention in general
- Face-to-face appointment week 78
  - Participant measures body weight, non-fasting blood glucose and blood pressure himself/herself
  - Follow up of weight loss and weight maintenance, blood sugar, blood pressure and the intervention in general
- Face-to-face appointment week 82
  - Participant measures body weight, non-fasting blood glucose and blood pressure himself/herself
  - Follow up of weight loss and weight maintenance, blood sugar, blood pressure and the intervention in general
- Face-to-face appointment week 86
  - Participant measures body weight, non-fasting blood glucose and blood pressure himself/herself
  - Follow up of weight loss and weight maintenance, blood sugar, blood pressure and the intervention in general
- Face-to-face appointment week 90
  - Participant measures body weight, non-fasting blood glucose and blood pressure himself/herself
  - Follow up of weight loss and weight maintenance, blood sugar, blood pressure and the intervention in general
- Face-to-face appointment week 94
  - Participant measures body weight, non-fasting blood glucose and blood pressure himself/herself
  - Follow up of weight loss and weight maintenance, blood sugar, blood pressure and the intervention in general
- Face-to-face appointment week 98
  - Participant measures body weight, non-fasting blood glucose and blood pressure himself/herself
  - Follow up of weight loss and weight maintenance, blood sugar, blood pressure and the intervention in general
- Face-to-face appointment week 102
  - Participant measures body weight, non-fasting blood glucose and blood pressure himself/herself
  - Follow up of weight loss and weight maintenance, blood sugar, blood pressure and the intervention in general

Supplemental material 6 Support for the eHealth support group and the face-to-face support group during the eHealth DIabetes remission Trial (eDIT)

		Total diet replacement phase							Food reintroduction phase				Weight maintenance phase																					
	Week	0	1	2	4	6	8	10	12	14	16	18	20	22	26	30	34	38	42	46	50	54	58	62	66	70	74	78	82	86	90	94	98	102
**eHealth support group**	Scheduledvideo meetings	x		x		x			x	x	x	x	x		x			x				x												
	HbA1c measure at home	x					x				x						x		x		x		x		x		x		x		x		x	
	Daily home measures of body weight, fasting glucose and blood pressure																																	
**Face-to-face support group**	Face-to-face meetings at the clinic	x	x	x	x	x	x	x	x	x	x	x	x	x	x	x	x	x	x	x	x	x	x	x	x	x	x	x	x	x	x	x	x	x
	Body weight, non-fasting glucose and blood pressure measure at the clinic	x	x	x	x	x	x	x	x	x	x	x	x	x	x	x	x	x	x	x	x	x	x	x	x	x	x	x	x	x	x	x	x	x

Supplemental material 7 Consent form

Samtycke till att delta i projektet

Jag har fått muntlig och/eller skriftlig information om studien och har haft möjlighet att ställa frågor. Jag får behålla den skriftliga informationen.

- Jag samtycker till att delta i projektet *Remission av typ 2 diabetes med hjälp av eHälsa (eDIT)*
- Jag samtycker till att mina prover sparas i en biobank på det sätt som beskrivs i forskningspersonsinformationen.

Plats och datum	Underskrift
	Namnförtydligande

Samtycke till framtida forskning

Jag har fått information om att de prover jag lämnar kan bli aktuella för framtida forskning som inte är beskriven i informationen till mig som forskningsperson. Jag har även fått information om att i det fall mina prover ska användas i framtida forskning måste Etikprövningsmyndigheten göra en prövning av det nya projektet och i sin prövning avgöra om jag ska tillfrågas på nytt.

- Jag samtycker till att mina prover får sparas för framtida forskning.

Plats och datum	Underskrift
	Namnförtydligande
