# Supplementary material for: eHealth versus face-to-face support for remission of type 2 diabetes by calorie restriction (eHealth DIabetes remission Trial): study protocol for a non-inferiority parallel group randomised controlled trial
Source: BMJ Open. 2025 Jul 22;15(7):e095100. doi: 10.1136/bmjopen-2024-095100 (PMC12306309; doi:10.1136/bmjopen-2024-095100)
Supplement: Supplementary file 2 [file bmjopen-2024-095100.reviewer_comments.pdf]

PEER REVIEW HISTORY

BMJ Open publishes all reviews undertaken for accepted manuscripts. Reviewers are asked to complete a checklist review form and are provided with free text boxes to elaborate on their assessment. These free text comments are reproduced below.

ARTICLE DETAILS

Title (Provisional)

eHealth vs face-to-face support for remission of type 2 diabetes by calorie restriction (eDIT): study protocol for a non-inferiority parallel group randomized controlled trial

Authors

Otten, Julia; Tellström, Anna; Schien, Claudia; Chninou, Youssef; Lindholm, Lars; Winkvist, Anna; Liv, Per; Stomby, Andreas

---

VERSION 1 - REVIEW

**Reviewer**	**1**
**Name**	**Cho, Kyu Yong**
**Affiliation**	**Hokkaido University, Division of Diabetes and Obesity, Faculty of Medicine and Graduate School of Medicine**
**Date**	**13-Feb-2025**
**COI**	**None**

Dear Authors,

I was pleased to review your submitted manuscript. You have outlined a protocol for a two-arm RCT to demonstrate that eHealth support is non-inferior to in-person support in assisting patients with type 2 diabetes in achieving remission through calorie restriction. The results are promising and could be clinically useful. To improve reader understanding, I have a few suggestions:

1. Please describe how you will ensure that subjects in the eHealth support group adhere to the proposed interventions and recommendations.

2. The non-inferiority margin of 0.8% appears quite large compared to the more commonly used margins of 0.3-0.4%. Please thoroughly justify the appropriateness of this margin and provide further explanation if needed.

**Reviewer**	**2**
**Name**	**Saisho, Yoshifumi**

Affiliation **Keio University School of Medicine Graduate School of Medicine Division of Rheumatology Department of Internal Medicine**

Date **09-Jun-2025**

COI **None**

The study protocol is carefully designed. The purpose of this protocol is to test the hypothesis that an eHealth program will be non-inferior to face-to-face meetings in helping patients with T2D achieve remission through caloric restriction.

Now we have a novel therapeutic modality, tirzepatide. This study protocol should include tirzepatide in a list of anti-diabetic medications.

Also, the combination of caloric restriction and tirzepatide may increase the success rate of remission. The authors may mention and discuss this point in the protocol.

VERSION 1 - AUTHOR RESPONSE

Reviewer: 1
Dr. Kyu Yong Cho, Hokkaido University

Comments to the Author:
Dear Authors,
I was pleased to review your submitted manuscript. You have outlined a protocol for a two-arm RCT to demonstrate that eHealth support is non-inferior to in-person support in assisting patients with type 2 diabetes in achieving remission through calorie restriction. The results are promising and could be clinically useful. To improve reader understanding, I have a few suggestions:

1. Please describe how you will ensure that subjects in the eHealth support group adhere to the proposed interventions and recommendations.
We have added additional information on page 6 how the study team ensures adhearance for the eHealth support group.

2. The non-inferiority margin of 0.8% appears quite large compared to the more commonly used margins of 0.3-0.4%. Please thoroughly justify the appropriateness of this margin and provide further explanation if needed.
We have added further justification for our chosen non-inferiority margin of 0.8% to the Methods section (page 9). This margin was determined prior to the start of the trial in 2022. We acknowledge that most pharmaceutical trials in the diabetes field commonly use a smaller margin of 0.3–0.4%. However, as participant recruitment began in August 2022, we have chosen not to alter the non-inferiority margin retrospectively. That said, we have now explicitly acknowledged the use of a wider margin (0.8% instead of 0.4%) as a study limitation, and this has been added to the Limitations section at the beginning of the manuscript.

Reviewer: 2
Dr. Yoshifumi Saisho, Keio University School of Medicine Graduate School of Medicine Division of Rheumatology Department of Internal Medicine

Comments to the Author:
The study protocol is carefully designed. The purpose of this protocol is to test the hypothesis that an eHealth program will be non-inferior to face-to-face meetings in helping patients with T2D achieve remission through caloric restriction.

Now we have a novel therapeutic modality, tirzepatide. This study protocol should include tirzepatide in a list of anti-diabetic medications.

Tirzepatide has been added to the list of antidiabetic medication provided in Supplementary Material 2.

Also, the combination of caloric restriction and tirzepatide may increase the success rate of remission. The authors may mention and discuss this point in the protocol.
We agree that tirzepatide is a highly effective treatment for weight loss, and we have added a discussion on this to page 5 of the manuscript. In the present study, we have chosen to avoid the use of GLP1 receptor agonists and tirzepatide, as their administration would preclude the ability to accurately assess diabetes remission. However, if blood glucose levels rise, participants are reinitiated on glucose-lowering medications, and both GLP1 receptor agonists and tirzepatide are considered as treatment options. Recruitment for this study began in 2022, and we have since observed a marked shift in patient preferences. Compared to 2022, there is now considerably greater interest in combining calorie restriction with GLP1 receptor agonists or tirzepatide. Nonetheless, such combination therapy is not permitted within the current study protocol."
